# A cross-sectional assessment to detect type 2 diabetes with endothelial and autonomic nervous system markers using a novel system

**DOI:** 10.1186/s40200-014-0118-x

**Published:** 2014-12-14

**Authors:** John E Lewis, Laura Lantigua, Steven E Atlas, Johanna Lopez, Armando Mendez, Sharon Goldberg, Sacha Medici, Janet Konefal, Judi M Woolger, Eduard Tiozzo, Karyem H Aliffe

**Affiliations:** Department of Psychiatry & Behavioral Sciences, University of Miami Miller School of Medicine, 1120 NW 14th Street, Suite #1474 (D28), Miami, FL 33136 USA; Department of Medicine, University of Miami Miller School of Medicine, Miami, FL 33136 USA; Division of Hospital Medicine, University of Miami Miller School of Medicine, Miami, FL 33136 USA; Pharmacognosia, Rainier, WA 98576 USA

**Keywords:** Type 2 diabetes mellitus, Diabetic autonomic neuropathy, Autonomic nervous system, Cardiometabolic risk, Glycosylated hemoglobin, Oral glucose tolerance test

## Abstract

**Background:**

Type 2 diabetes mellitus is frequently unrecognized until complications appear. Diabetic autonomic neuropathy is one of the early complications of type 2 diabetes mellitus, resulting in autonomic nervous system (ANS) dysfunction. The purpose of this study was to determine the validity of ANS function indicators to screen for type 2 diabetes mellitus, as measured by the TM-Oxi and SudoPath system.

**Methods:**

All enrolled participants completed a basic sociodemographic and medical history questionnaire including current medications. Healthy controls (n = 25) underwent a 2-hour oral glucose tolerance test (OGTT) to evaluate glucose, insulin, and insulin C-peptide. Patients with type 2 diabetes mellitus (n = 24) were assessed with fasting plasma glucose (FPG) and glycosylated hemoglobin. The TM-Oxi and SudoPath system evaluation was completed by all subjects. Data were analyzed using SPSS 22. Frequency and descriptive statistics were calculated on all variables. The criterion for statistical significance was α = 0.05.

**Results:**

The twenty-five healthy controls had a mean age of 37.0 years. The twenty-four type 2 diabetes mellitus patients currently undergoing standard treatment had a mean age of 48.9 years. Based on the American Diabetes Association guidelines, we detected pre-diabetes in 4 subjects and diabetes in 1 subject, while all other subjects had normal FPG values. At 120 minutes, the correlations between the OGTT and cardiometabolic risk score (CMRS) were: r = 0.56 (p = 0.004) for glucose and r = 0.53 (p = 0.006) for insulin. At 120 minutes, the correlations between the OGTT and photoplethysmography index (PTGi) were: r = -0.56 (p = 0.003) for glucose and r = -0.41 (p = 0.04) for insulin. The CMRS, PTGi, and plethysmography total power index (PTGVLFi) differed significantly between the diabetes patients and healthy participants. The specificity and sensitivity for the CMRS, PTGi, and PTVLFi comparing the diabetes patients with healthy controls were high.

**Conclusion:**

The TM-Oxi and SudoPath system shows promise as a valid, convenient, and non-invasive screening method for type 2 diabetes mellitus. The ANS function and CMR indicators measured by this system may be useful in guiding diabetes and cardiovascular health screening, treatment, and monitoring.

## Introduction

The incidence of type 2 diabetes mellitus has increased in recent decades to epidemic proportions. As of 2010, 25.8 million people in the U.S. were estimated to have diabetes (90-95% of the cases were type 2 diabetes mellitus) [1], and according to the Centers for Disease Control and Prevention, one in three children born in the year 2000 will eventually become diabetic [2,3]. Because the complications of diabetes insidiously damage multiple organ systems, type 2 diabetes mellitus has become a major public health threat [4].

Early detection of diabetes is warranted for preventing disease progression and its associated macrovascular (coronary artery disease, peripheral arterial disease, and stroke) and microvascular complications (diabetic nephropathy, neuropathy, and retinopathy) [1,3,5]. One of the poorly understood yet serious and most common complications of type 2 diabetes mellitus is diabetic autonomic neuropathy (DAN) [6]. DAN can cause dysfunction of the autonomic nervous system (ANS), which controls autonomic body functions through the balanced opposition of the parasympathetic (PNS) and sympathetic nervous systems (SNS) [6]. Diabetes-associated metabolic disturbances (e.g., hyperglycemia) can lead to neuro-hormonal growth deficiency in the ANS through deregulated cell signaling pathways, direct neuronal damage, reduced neuronal blood flow, increased oxidative stress, and altered nitric oxide (NO) homeostasis [7,8].

One of the clinical manifestations of DAN occurs in the sudomotor system. The SNS controls sweat production in the skin, but in patients with diabetes who also have DAN, ANS dysfunction results in loss of sweating and dry skin. Dry skin cracks, providing portals of entry for microorganisms, which can lead to infectious ulcers, gangrene, and amputation [8-10]. Other organ systems, including the cardiovascular, gastrointestinal, genitourinary, and ocular, are also susceptible to ANS dysfunction in type 2 diabetes mellitus [8,11].

Cardiovascular autonomic neuropathy (CAN), a form of DAN, is an early and frequent complication of type 2 diabetes mellitus that may occur within one year of diagnosis in up to 20% of patients with diabetes [8,12,13]. CAN is a consequence of damage to autonomic nerve fibers that innervate the heart and cardiac blood vessels, resulting in abnormalities in heart rate (i.e., reduced heart rate variability) and vascular dynamics (i.e., abnormal blood flow, volume, and pressure) [8,14]. Both DAN and CAN develop silently and slowly over years and represent early complication of type 2 diabetes mellitus.

Autonomic function tests can detect ANS dysfunction at an early stage in asymptomatic type 2 diabetes mellitus patients [8,14]. In the early 1970s, several noninvasive cardiovascular reflex tests were proposed to assess cardiovascular autonomic function in order to diagnose CAN [8,15]. Those tests included the Valsalva maneuver, deep breathing, and heart rate and blood pressure response to standing up. Furthermore, ANS function in the sudomotor system can be assessed by measuring the sympathetic skin response generated on the sweat glands and the overlying epidermis [8]. Thus, the TM-Oxi and SudoPath system was designed to evaluate ANS function by measuring changes in heart rate, vascular dynamics, and skin response during the performance of those autonomic function tests. The measurements are integrated with anthropometric, physical activity, and endothelial function data in software to calculate various cardiovascular ANS function indicators, such as a photoplethysmographic (PTG) very low frequency index (PTGVLFi), a PTG index (PTGi), and a cardiometabolic risk score (CMRS).

ANS dysfunction and CMR indicators calculated by the TM-Oxi and SudoPath system may be effective in detecting pre-clinical disease and improving the management of type 2 diabetes mellitus [5,8]. Existing screening, diagnostic, and monitoring methods for type 2 diabetes mellitus are all invasive, time consuming, and involve some level of discomfort to the participant. Furthermore, approximately one-third of the individuals with type 2 diabetes mellitus are undiagnosed [3]. Therefore, a rapid, non-invasive, and inexpensive screening device such as the TM-Oxi and SudoPath system may facilitate early detection and improve management of the disease. The purpose of this study was to determine the validity of ANS function and CMR indicators, calculated by the TM-Oxi and SudoPath system, in screening for type 2 diabetes mellitus.

## Methods

### The TM-Oxi and SudoPath system

**Figure 1:**
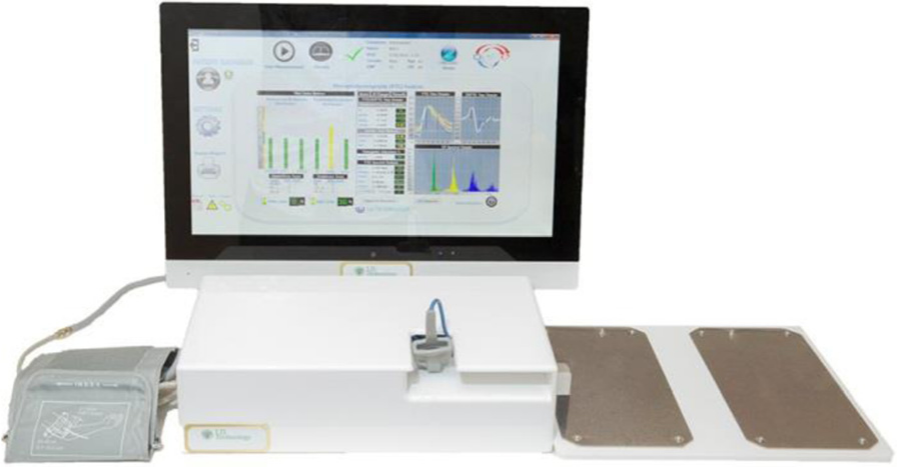
**Photograph of the TM-Oxi and SudoPath system.** This machine is a software complex managing three FDA cleared devices; a blood pressure device, an oximeter, and a galvanic skin response device.

The TM-Oxi assesses cardiac sympathetic and parasympathetic ANS function using an automatic oscillometric blood pressure device and a pulse oximeter (Figure 1). The pulse oximeter device uses an optical technique to measure changes in vascular dynamics and heart rate. Briefly, light of a suitable wavelength that is directed into the nail bed area of the right index finger is absorbed, reflected, and scattered by blood hemoglobin. During systole, the arterial diameter increases as blood fills the fingertip capillary beds, and the presence of more hemoglobin in the vessels alters the absorption, reflection, and scattering of the light, measured by a photosensor. This creates a pulsatile signal or waveform illustrated as a PTG that varies in time with each heartbeat. The PTG is used to analyze heart rate variability (HRV), defined as the variation in time intervals between each heart beat (i.e., R-R interval) that results from PNS and SNS activity on the heart’s sinus node [16,17].

**Figure 2:**
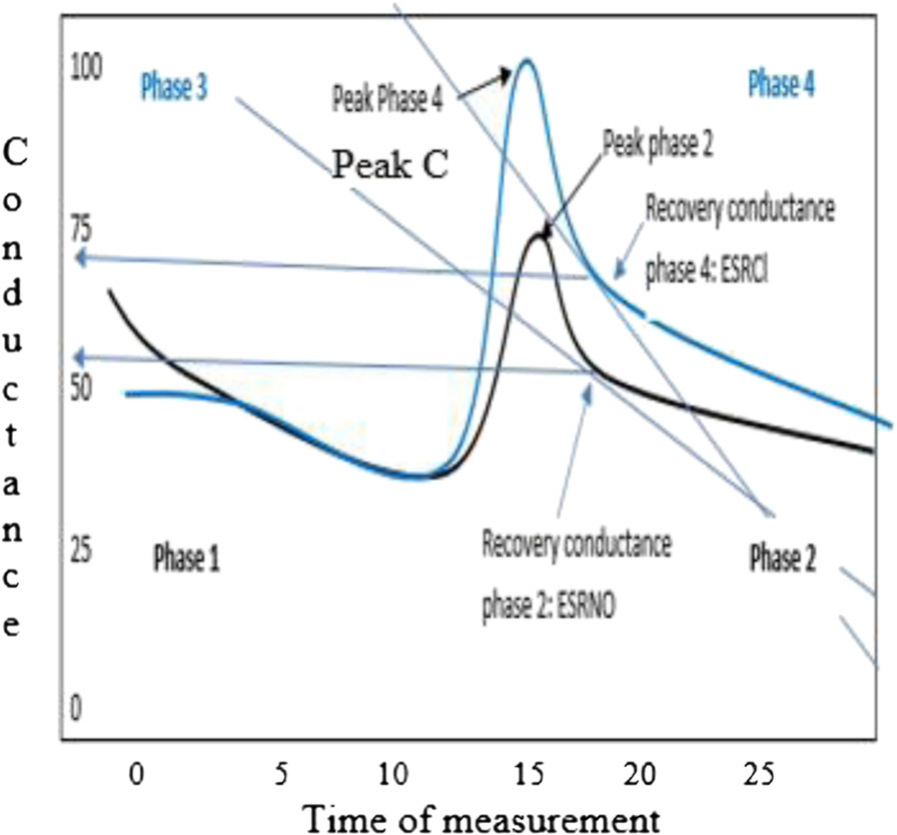
**Current peaks during electrical stimulation of the skin.** The blue current peak corresponds to hyperemia, and the white current peak corresponds to chloride release via the sweat glands. In the ordinate, current peaks in conductance are expressed in micro-siemens, and the abscissa is expressed in time in seconds.

The TM-Oxi software uses the PTG to analyze spontaneous HRV and of experimentally induced fluctuations of the R-R intervals during cardiovascular autonomic reflex tests (i.e., Valsalva maneuver, deep breathing, and change in posture) [18], as discussed at the manufacturer’s website (http://www.ldteck.com/endothelial-dysfunction.html). The patented signal processing and analysis of the TM-Oxi (Patent PCT/IB2013/002595) applies a mathematical algorithm, the fast Fourier transform (FFT), to the PTG recording (FFTPTG) to analyze the three frequency components of HRV (Figure 2): (a) a very low frequency (PTGVLF) component associated with thermoregulation and sweating due to oscillations in vasomotor tone (sympathetic control); (b) a low frequency (PTGLF) component related to the baroreflex, which is under sympathetic control with parasympathetic modulation; and (c) a high frequency (PTGHF) component associated with R-R changes during the phases of breathing that is under parasympathetic control. Parameters calculated from these components include: (a) PTG total power (PTGTP), which is the sum of the areas under the curve covered by the 3 frequency components; (b) PTGi, which is the sum of the amplitudes of the 3 frequency components; and (c) PTGVLFi, which is the ratio between PTGVLF and the electric skin response to nitric oxide (ESRNO), a marker related to skin blood flow, derived from the SudoPath (described below), as detailed at the manufacturer’s website (http://www.ldteck.com/endothelial-dysfunction.html).

Besides assessing autonomic function, PTG also provides information about endothelial function and arterial stiffness. The PTG signal is also quantized into a digital volume pulse (DVP) waveform that represents the pressure wave that propagates from the heart to the periphery (i.e., systolic peak) and reflection back to the heart (i.e., diastolic peak) during the cardiac cycle [19-21]. Arterial stiffness, partially as a result of endothelial dysfunction, is defined as the arterial opposition to the pressure wave, resulting in abnormal systolic and diastolic peaks. Thus, the DVP contour, although affected by body temperature and perfusion, is mainly determined by myocardial and arterial characteristics, and it is used by the TM-Oxi to calculate variables (i.e., systolic/diastolic peaks, reflection index, and stiffness index) that are indicators of vascular health and arterial status. Finally, PTG is used by the TM-Oxi to calculate the CMRS. The CMRS is calculated using the following variables from the TM-Oxi: systolic and diastolic blood pressure, body mass index (BMI), physical activity level, endothelial function, and ANS function indicators. Each variable is scored as 0 = normal, 1 = borderline, or 2 = abnormal to calculate the CMRS.

**Figure 3:**
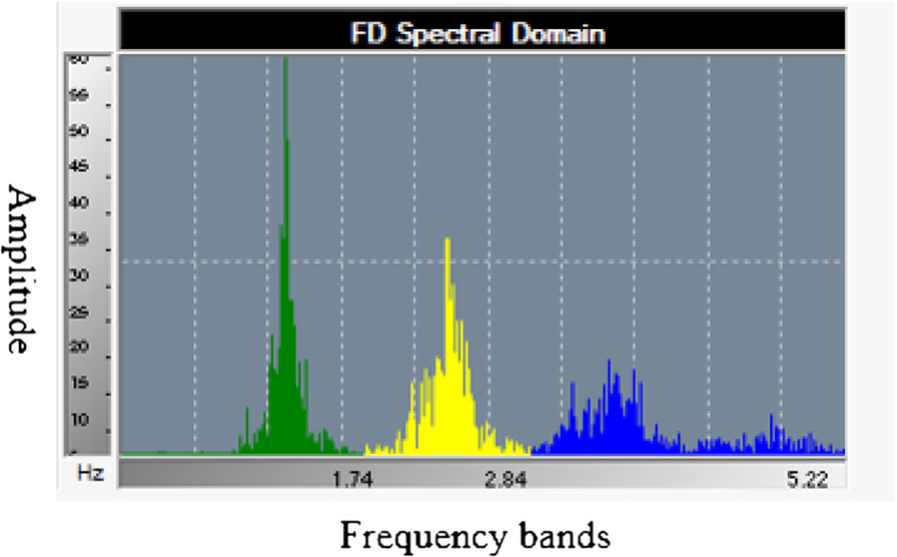
**Fast Fourier transform (FFT) of a photoplethysmograph waveform.** The waveform in the frequency domain is separated into three frequency bands: the ‘very low frequency or PTGVLF’ (in green), the ‘low frequency or PTGLF’ (in yellow), and the ‘high frequency or PTGHF’ (in blue). All area measurements are expressed in millisecond square (ms^2^). In the ordinate, the FFT amplitude is in millivolts (mV), and in the abscissa the band of frequency is in Hertz (Hz).

The SudoPath uses two stainless steel electrodes placed under the soles of the feet, where sweat gland density is very high. It assesses galvanic skin response by measuring the electrical conductance of the skin, which is dependent on the amount of sweat-induced moisture, and hence, is indicative of SNS function. The patented device (US patent number 61835064) generates a low voltage signal with weak DC current that is fed to the active electrode, passing through the interstitial fluid and reaching the skin in contact with the passive electrode. The current provokes an electrical stimulation of the post-sympathetic cholinergic fiber, which releases acetylcholine, stimulating nicotinic muscarinic receptors (M-receptors) in the skin and sweat glands [22]. In endothelial cells, M-activation results in increased NO that causes relaxation and vasodilation in adjacent vascular smooth muscle, thus increasing blood flow. In the sweat gland, activation of M-receptors promotes chloride movement across the apical membrane of the sweat gland cells, depolarizing it to generate a negative potential that drives sodium and water into the lumen for sweat production [23]. The change in sweat production and blood flow affects the electrical conductance of the skin, which is measured by the device. Figure 3 represents the measured conductance provoked by the electrochemical reaction of water and chloride ions on the bulk of the electrodes, which is measured as ESRNO. This method is fully described at the manufacturer’s website (http://www.ldteck.com/galvanic-skin-response.html).

### Study participants

The study was conducted with the approval of the University of Miami Institutional Review Board for human subjects research. Each participant signed informed consent and HIPAA forms prior to study entry. Potential participants (n = 80) were identified through referrals at the University of Miami Miller School of Medicine from April 2013 to February 2014. Of 55 eligible participants, 50 were enrolled in the study, and the other 5 decided not to participate. One type 2 diabetes mellitus patient was eliminated from the subsequent data analysis because of a poor quality signal from the TM-Oxi.

### Inclusion and exclusion criteria

Inclusion criteria were: (a) 18+ years of age; (b) English or Spanish speaking; (c) ability to provide written informed consent; and (d) diagnosed with type 2 diabetes mellitus or healthy with no known chronic diseases or disorders. Exclusion criteria were: (a) diagnosis of type 1 diabetes, HIV/AIDS, active cancer and/or current chemotherapy or radiation treatment, hypothyroidism, atrioventricular block, Raynaud’s disease, Alzheimer’s disease, or Parkinson’s disease; (b) current use of alpha or beta blockers or corticosteroids; (c) contraindications to the use of the TM-Oxi and SudoPath system, including presence of an automatic implantable defibrillator, erratic, accelerated, or mechanically-controlled irregular heart rhythms, arterial fibrillation/flutter, or any implanted electronic device; or (d) an acrylic nail or nail polish on the right index finger.

### Outcomes and assessments

All enrolled participants completed a basic sociodemographic and medical history questionnaire including current medications. All participants were instructed to fast for a minimum of 8 hours prior to blood sampling. Healthy subjects underwent a two-hour OGTT to evaluate glucose, insulin, and insulin C-peptide. Type 2 diabetes mellitus patients were assessed with venous fasting plasma glucose (FPG) and glycosylated hemoglobin (GHb). The TM-Oxi and SudoPath system evaluation was completed by all subjects.

### Statistical analysis

Data were analyzed using SPSS 22 (IBM Inc., Chicago, IL) for Windows. Frequency and descriptive statistics were calculated on all variables. Independent-sample *t*-test and chi square were utilized to determine the presence of differences in background contextual variables by group assignment. For healthy subjects, we evaluated the OGTT results at 120 minutes for the detection of pre-diabetes and diabetes according to standard criteria. We used Pearson product-moment correlations between the CMRS and PTGi and OGTT values. Healthy subjects and type 2 diabetes mellitus patients were compared with t-tests and receiver operating characteristic (ROC) curves to determine the specificity and sensitivity of the CMRS and PTGVLFi and PTGi as potential screening markers of diabetes. The criterion for statistical significance was α = 0.05.

## Results

### Safety and tolerability

No adverse events were reported with the use of the TM-Oxi and SudoPath system during the study.

### Sociodemographics and health variables

Table 1 displays the characteristics for each study group. Forty-nine subjects (n = 25 healthy controls and n = 24 patients with type 2 diabetes mellitus) were included in the study. The twenty-five healthy controls (12 males and 13 females) had a mean age of 37.0 years (SD = 17.5; R = 18, 78) and a mean BMI = 26.1 (SD = 5.6; R = 17.2, 43.6). The twenty-four type 2 diabetes mellitus patients currently undergoing standard treatment (12 males and 12 females) had a mean age of 48.9 years (SD = 11.0; R = 30, 72) and a mean BMI = 34.6 (SD = 8.3; R = 23.3, 56.1). The type 2 diabetes mellitus patients were significantly older, more likely to be black, and had a higher BMI, systolic blood pressure, and FPG (see Table 1). The duration of diagnosis of diabetes for the patients was: 10 or more years (40%; n = 10), 5 to 9 years (45%; n = 11), and less than 5 years (15%; n = 3). The type 2 diabetes mellitus patients had a mean GHb of 7.0% (53 mmol/mol) (SD = 1.3% (14.2 mmol/mol); R = 5.4%, 10.4% (36, 90 mmol/mol)). The primary treatments for the type 2 diabetes mellitus patients were: metformin (68%; n = 16), a secretagogue (28%; n = 7), insulin (5%; n = 1), and an antihypertensive (36%; n = 9).

**Table 1:** **Sociodemographic characteristics of the sample**

**Variable**	**Category**	**Diabetes patients (n = 24)**	**Healthy controls (n = 25)**	**Statistic, p value**
Age	-	48.9 ± 11.0 (30, 72)	37.0 ± 17.5 (18, 78)	t(47) = 2.8, p = 0.01
Gender	Male	12 (50%)	12 (48%)	Χ^2^(1) = 0.02, p = 0.89
	Female	12 (50%)	13 (52%)	
Race/Ethnicity	White, non-Hispanic	1 (4%)	11 (44%)	Χ^2^(4) = 19.3, p = 0.001
	Black, non-Hispanic	9 (38%)	0	
	Hispanic	13 (54%)	13 (52%)	
	Asian	1 (4%)	0	
	Middle Eastern	0	1 (4%)	
BMI (kg/m^2^)	-	34.6 ± 8.3 (23.3, 56.1)	26.1 ± 5.6; (17.2, 43.6)	t(47) = 4.2, p < 0.001
SBP (mm Hg)	-	138.1 ± 18.6 (102, 194)	120.4 ± 14.2 (4, 165)	t(47) = 3.8, p < 0.001
DBP (mm Hg)	-	79.6 ± 8.9 (63, 96)	75.1 ± 12.5 (52, 104)	t(47) = 1.5, p = 0.15
FPG (mg/dL)	-	131.7 ± 50.1 (79, 243)	85.2 ± 6.9 (72, 105)	t(47) = 4.6, p < 0.001

Note: Continuous data are represented by M ± SD (R); SBP = Systolic blood pressure SBP = Systolic blood pressure; DBP = Diastolic blood pressure; FPG = Fasting plasma glucose.

### Evaluation of diabetes risk among healthy subjects

Based on the American Diabetes Association guidelines, we detected pre-diabetes in 4 subjects (16%; values = 140, 143, 147, and 153 mg/dL) and diabetes in 1 subject (4%; value = 254 mg/dL), while all other subjects had normal glucose values (i.e., 139 mg/dL or less).

### Correlations between glucose, insulin, and insulin C-peptide from OGTT at 120 minutes with CMRS and PTGi

At 120 minutes, the correlations between the OGTT and CMRS were: r = 0.56 (p = 0.004) for glucose, r = 0.53 (p = 0.006) for insulin, and r = 0.58 (p = 0.002) for insulin C-peptide. At 120 minutes, the correlations between the OGTT and PTGi were: r = -0.56 (p = 0.003) for glucose, r = -0.41 (p = 0.04) for insulin, and r = -0.50 (p = 0.01) for insulin C-peptide.

### Comparisons between the healthy subjects and type 2 diabetes mellitus patients

The mean CMRS of the healthy subjects was 2.2 (SD = 2.9; R = 0, 9) and of the type 2 diabetes mellitus patients was 9.4 (SD = 3.7; R = 1, 14), and that difference was statistically significant (t = 7.6 (47), p < 0.001). The mean PTGi of the healthy subjects was 67.0 (SD = 25.0; R = 18.4, 123.3) and of the type 2 diabetes mellitus patients was 31.3 (SD = 11.5; R = 12.3, 68.5), and that difference was statistically significant (t = 6.4 (47), p < 0.001). The mean PTGVLFi of the healthy subjects was 27.7 (SD = 52.6; R = 3, 242) and of the type 2 diabetes mellitus patients was 61.8 (SD = 40.7; R = 12, 190), and that difference was statistically significant (t = 2.5 (47), p = 0.02).

### Receiver operating characteristic curves

**Figure 4:**
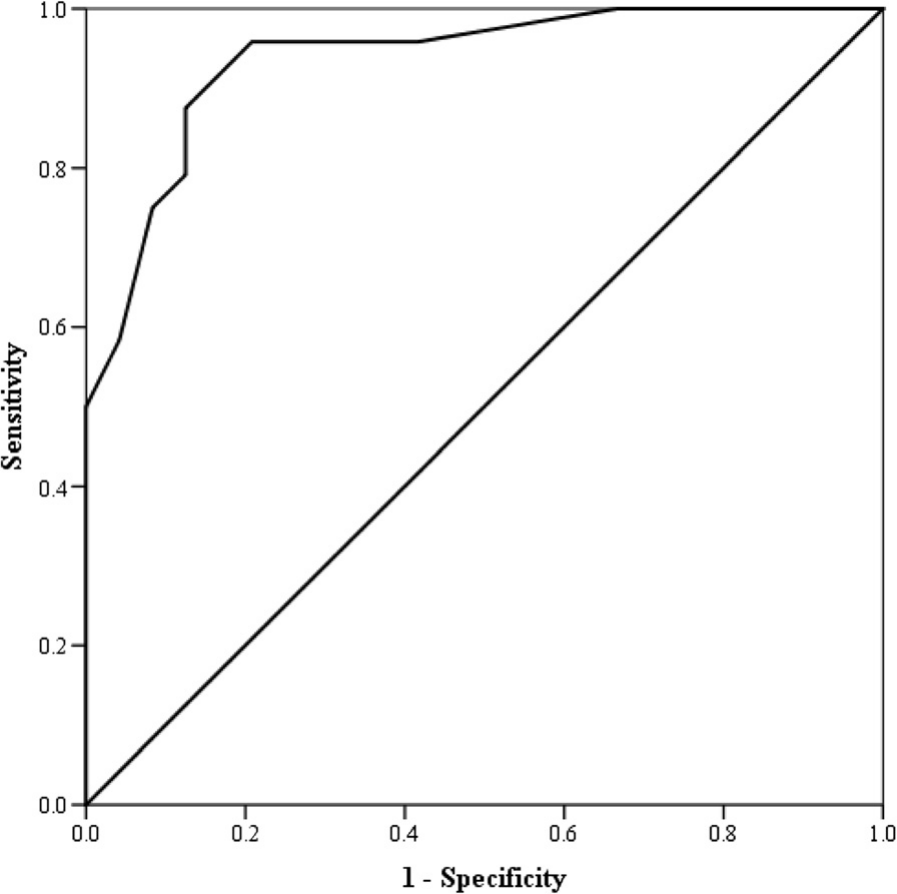
**Receiver operating characteristic curve for the cardiometabolic risk score.** The curve represents the sensitivity and specificity for the Cardiometabolic Risk Score.

**Figure 5:**
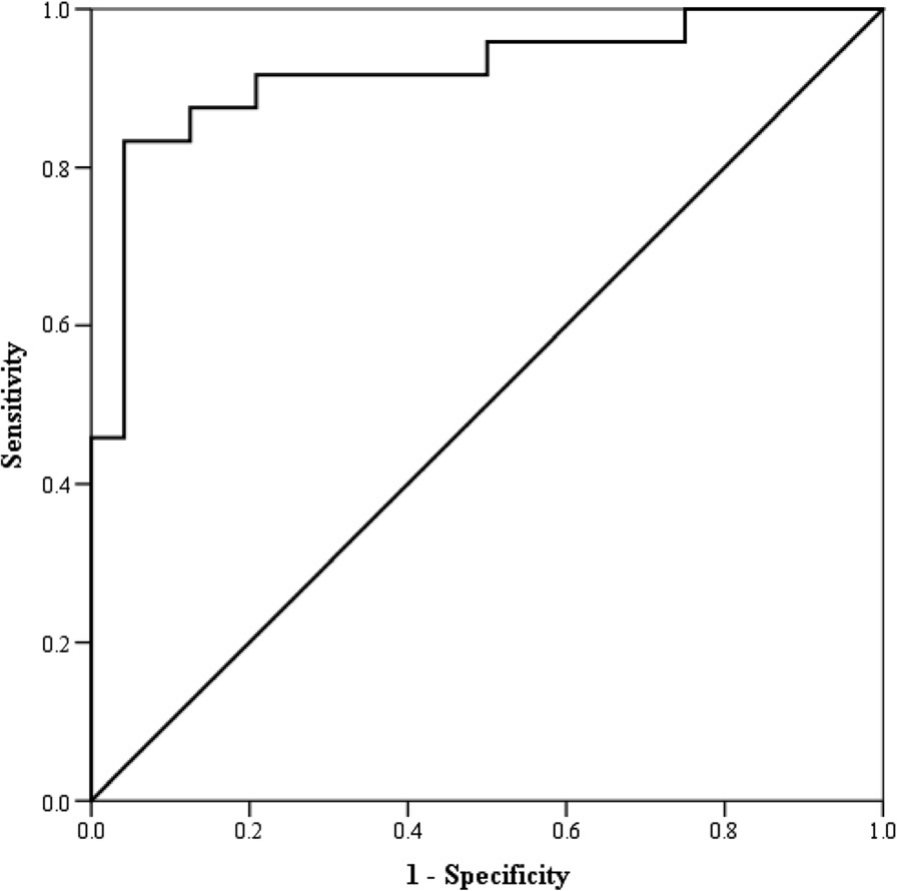
**Receiver operating characteristic curve for the photoplethysmographic index.** The curve represents the sensitivity and specificity for the Photoplethysmographic Index.

**Figure 6:**
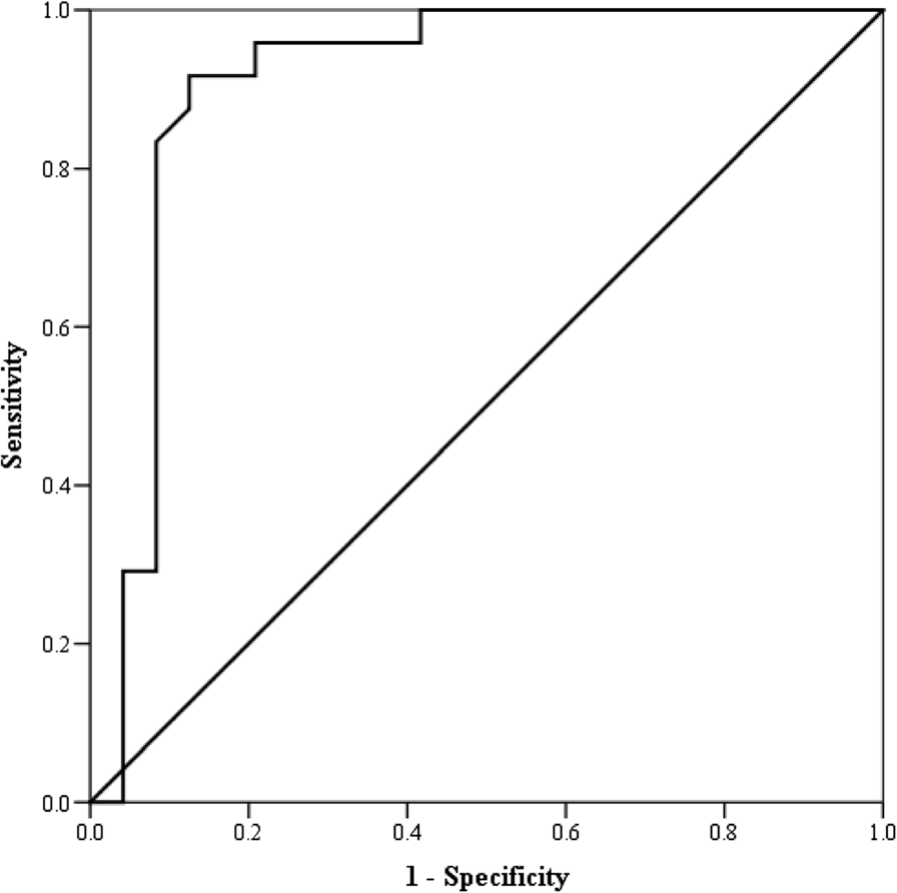
**Receiver operating characteristic curve for the photoplethysmographic very low frequency.** The curve represents the sensitivity and specificity for the Photoplethysmographic Very Low Frequency.

The CMRS had a sensitivity of 92% and specificity of 83% (cut-off score > 4) with an area under the curve = 0.94 (SE = 0.04; 95% CI = 0.87, 1.0) and an asymptotic significance < 0.001 (Figure 4). The PTGi had a sensitivity of 92% and specificity of 80% (cut-off score > 35.5) with the area under the curve = 0.92 (SE = 0.04; 95% CI = 0.84, 1.0) and an asymptotic significance < 0.001 (Figure 5). The PTGVLFi had a sensitivity of 92% and specificity of 87% (cut-off score > 25.5) with the area under the curve = 0.91 (SE = 0.05; 95% CI = 0.81, 1.0) and an asymptotic significance < 0.001 (Figure 6).

## Discussion

In our study, the TM-Oxi and SudoPath system was useful for detecting the presence of type 2 diabetes mellitus through its ability to assess ANS function. Currently used screening tests for type 2 diabetes mellitus are invasive, time-consuming, and have other disadvantages [24]. Although the 2-hour OGTT is the gold standard for diabetes testing [5], it is time consuming, not easily reproducible, affected by short-term lifestyle changes, and expensive [25]. For those reasons, the American Diabetes Association recommends screening for type 2 diabetes mellitus with FPG or GHb (FPG ≥ 126 mg/dl or GHb ≥ 6.5% or 48 mmol/mol) [3,26].

Nonetheless, FPG and GHb have their own limitations, including moderate sensitivity when compared to OGTT [24,27-29]. One population-based study of 2,753 participants showed that the correlations of FPG and GHb with OGTT were moderate at best (r = 0.46 and r = 0.33, respectively) [24]. When used as a sole screening test for type 2 diabetes mellitus, the sensitivity of FPG can be as low as 40%, whereas the specificity is about 84% [27]. Additionally, a multitude of reports have indicated that up to 50% of patients with diabetes who were diagnosed by OGTT criteria would have been missed by FPG criteria [30]. Measurement of GHb with a cut-point ≥ 6.5% or 48 mmol/mol is reasonably specific (> 88%), however the sensitivity ranges from 17% to 72.8% [28,29]. In our study, CMRS and PTGi variables demonstrated significant correlations with the 2-hour OGTT glucose. This is an improvement compared to the results of GHb and FPG in other research [24]. Furthermore, the CMRS, PTGi, and PTGVLFi were significantly different between type 2 diabetes mellitus and healthy participants. The specificity and sensitivity in differentiating patients with diabetes and healthy controls were high for each indicator. Thus, our results indicate the TM-Oxi and SudoPath system may prove useful as a screening tool for type 2 diabetes mellitus.

Another potential benefit of this system is to detect cardiovascular complications in the same patient population. The link between type 2 diabetes mellitus and CVD has been demonstrated in numerous studies [31]. CVD is listed as the cause of death in approximately 65% of individuals with type 2 diabetes mellitus. Individuals with diabetes not only face a higher mortality rate from CVD, but also a worse prognosis for survival than do CVD patients without diabetes. Detecting both the presence of diabetes and CVD development with the TM-Oxi and SudoPath system becomes relevant in reducing morbidity and mortality rates of these two intricately linked diseases. More importantly, the Subcommittee of the Toronto Consensus Panel on Diabetic Neuropathy recommends early screening of CAN for all patients with diabetes because it is often asymptomatic [18] and may have detrimental clinical consequences (e.g., silent myocardial infarction, ischemia, cardiorespiratory arrest, ventricular arrhythmia, left ventricular dysfunction, resting tachycardia, postural hypotension, and diabetic nephropathy, among others) [32,33].

Furthermore, endothelial dysfunction and arterial stiffness are associated with insulin resistance in type 2 diabetes mellitus [34,35]. The confluence of diabetes associated metabolic disturbances (i.e., hyperglycemia and dyslipidemia) with inflammatory mediators, platelets, and the vascular wall results in accelerated atherogenesis via endothelial dysfunction and arterial stiffening, leading to increased cardiovascular risk [20]. In our study, the measured indicators of endothelial dysfunction and arterial stiffness were used to calculate CMRS. These indicators along with CMRS could be used to assess the overall cardiovascular risk, in addition to those related to CAN, in type 2 diabetes mellitus patients.

## Conclusion

In sum, we have demonstrated that the TM-Oxi and SudoPath system may be useful in diabetes screening and treatment monitoring. Current standard testing, when applied to screening large populations of asymptomatic individuals, and monitoring those who have been positively identified as having type 2 diabetes mellitus can be costly [27]. Furthermore, the demand is increasing for the development of low-cost, simple, and portable technologies to be used in the community and clinical setting. The TM-Oxi and SudoPath assessment is also non-invasive, which is relevant to approximately 10% of individuals in medical settings who have an excessive fear of needles that can result in avoidance and distress. Finally, the assessment is quick to perform, since it only takes approximately 10 minutes to complete, and the results are available immediately.

## Limitation of study

Limitations of our study include a relatively small sample size, a disproportionately greater number of Caucasians in the healthy control group, and a significant difference in average age between groups. Additionally, the OGTT was not administered on type 2 diabetes mellitus patients, and GHb was not measured in the healthy subjects. Nonetheless, our study demonstrated that the TM-Oxi and SudoPath system shows promise as a screening tool for type 2 diabetes mellitus. The ANS function and indicators measured by this system may be useful in guiding diabetes and cardiovascular health screening, treatment, and monitoring. Larger scale studies are warranted to substantiate these results and explore the clinical and prognostic significance of the scoring system and various indicators.

## Abbreviations

ANS: Autonomic nervous system
BMI: Body mass index
CAN: Cardiovascular autonomic neuropathy
CMRS: Cardiometabolic risk score
CVD: Cardiovascular disease
DAN: Diabetic autonomic neuropathy
DVP: Digital volume pulse
ESRNO: Electric skin response to nitric oxide
FFT: Fast Fourier transform
FFTPTG: Frequency spectrum of PTG data
FPG: Fasting plasma glucose
GHb: Glycosylated hemoglobin
HRV: Heart rate variability
NO: Nitric oxide
OGTT: Oral glucose tolerance test
PNS: Parasympathetic nervous system
PTG: Photoplethysmograph
PTGHF: Component associated with R-R changes during the phases of breathing that is under parasympathetic control
PTGLF: A low frequency component related to baroreflex
PTGi: Photoplethysmography index
PTGTP: Plethysmography total power index
PTGVLF: Very low frequency component of PTG data
ROC: Receiver operating characteristic
SNS: Sympathetic nervous system
